# Validity of postmortem computed tomography for use in forensic odontology identification casework

**DOI:** 10.1007/s12024-023-00591-9

**Published:** 2023-03-16

**Authors:** Sharon Maley, Denice Higgins

**Affiliations:** Forensic Odontology Unit, The University of Adelaide, Adelaide, SA 5005 Australia

**Keywords:** Forensic science, Forensic odontology, Human identification, Postmortem computed tomography

## Abstract

Forensic Odontology (FO) identification compares antemortem (AM) and postmortem (PM) dental datasets and is widely accepted as a primary identifier. Traditionally, a PM dental examination is undertaken in the same manner as a dental examination conducted for a living patient. Recently, the increased forensic application of computed tomography (CT) offers an alternative source of PM data. While charting from PMCT is widely accepted as less accurate, the impact on reconciliation is unknown. This study aims to determine if reconciliation outcome differs when PM dental data is collected from PMCT, compared with conventional PM examination. PMCT data was reviewed for 21 cases previously completed using conventional PM dental examination. Operators blinded to original identification outcomes charted from CT images before comparing to AM data to form an opinion regarding identity. Opinions formed were compared with original identification outcomes. Differences in PM dental charting between the two methods and the evidentiary value of AM and PM datasets were assessed to determine driving factors of differences in identification outcome. Compared to conventional PM dental examination, PMCT examination resulted in similar or less certain identification outcomes. Discrepancies in outcome were driven by the quality of AM and PM datasets rather than inaccuracies in charting from PMCT. Based on the results of this study, both conventional and PMCT methods of PM dental examination can reach similar identification outcomes. However, operators remained more certain in establishing identity when conducting conventional PM dental examinations especially when AM data was lacking.

## Introduction

Human identity is widely recognized as an essential human right, as is the right to a name at death [1]. Human identification is a crucial function of the forensic sciences since identity is vital to society and a legal requirement in many jurisdictions [2]. Numerous methods can be used to identify individuals, but only primary methods are accepted as standalone identifiers. Primary identification methods include DNA, fingerprints, and dental data comparison. Dental comparison is often used to identify burnt, decomposed, skeletonized, or fragmented remains due to tooth enamel’s hardness, which allows it to better survive decomposition, immersion, extreme heat, desiccation, or trauma when compared with soft tissues [3].

Identification cases in forensic odontology (FO) involve the comparison of antemortem (AM) and postmortem (PM) dental data. The reconciliation of individualizing characteristics of the dentition described in both AM and PM data allows FO practitioners to form an opinion on the likelihood that the two datasets pertain to the same individual [3]. This likelihood is expressed utilizing the INTERPOL scale, which allows exclusion or varying levels of certainty for identification [4]. To facilitate this process, both datasets are charted on an odontogram (a pictorial representation of the dentition) using standardized three-letter codes. AM data is information collated from dental records created throughout a person’s life, which includes clinical notes and radiographs. PM data is information collected by the examination of the jaws and teeth of a deceased person. Traditionally, PM examinations mimic those conducted on living patients with direct visual and tactile assessments as well as photographic and radiographic image creation. Recent advances in computed tomography (CT) in forensic science have provided an alternative or additional source of PM dental information.

The first use of CT for forensic purposes was in 1977 when forensic pathologists analyzed a CT scan to describe a gunshot wound to the head [5]. PMCT images have gained increasing recognition since this initial application, with many forensic institutions now routinely capturing PMCT data which is widely used to aid traditional autopsy [6–8]. PMCT images have grown in popularity because they offer rapid 3-dimensional imaging in a digital format with minimal disruption to human remains. This format also allows remote analysis and archiving of PM information for future assessment [5]. The advantages of PMCT and the increasing frequency of its acquisition make it reasonable to expect that, in the future, the analysis of PMCT for FO identification will become routine. Potentially, PMCT analysis can augment or even replace conventional PM dental examinations in FO identification casework.

Recent reports have emphasized the importance of a strong scientific basis for legal acceptance of expert opinion evidence [9, 10]. Hence, new technologies used in the formation of expert opinion require proven scientific validity before acceptance [11]. This has not yet been achieved for the use of PMCT in forensic odontology identification. Leow and Higgins (2020) found FO practitioners lacked confidence in the accuracy of PM charting when using only 2D images reconstructed from PMCT compared with conventional radiographs [12]. Another study by Ruder et al. (2016) showed the comparison of AM radiographs with PMCT can be a reliable method of identification in some instances but did not compare with conventional PM examination [13]. Several other studies have compared the accuracy of charting from PMCT with that from conventional examinations [14–16]. In general, these researchers found that using only PMCT led to inaccuracies in charting and recommended that it should not be used alone when collecting PM dental information. Although these previous assessments suggest that dental charting from PMCTs is less accurate and detailed than conventional PM dental examinations, none investigate whether these differences impact identity outcomes.

Therefore, this study examines whether reconciliation outcomes differ when PM dental data is collected using only CT imaging or conventional dental examinations. This study will contribute to a greater understanding of the utility of PMCT for FO identification.

## Materials and methods

The study has been approved by the Human Research Ethics Committee of the University of Adelaide (Ethics approval no.: H-2016–189) and is deemed to meet the requirements of the National Statement on Ethical Conduct in Human Research 2007 (updated 2018). This is a retrospective study utilizing FO identification cases previously completed by the Forensic Odontology Unit of the University of Adelaide between October 2020 and January 2021. The Coroner of South Australia has given approval for the use of deidentified data from completed FO identifications in casework validation studies.

The inclusion criteria for cases were that identification had been previously determined using only conventional PM examination methods providing a “gold standard” outcome, PMCT data was available and not utilized during original case completion, and operators were blinded to original casework identification outcomes and not involved in original casework completion. A final sample size of 21 cases was obtained. PMCT scans were performed at Forensic Science SA (FSSA) using a Siemens SOMATOM Definition Edge CT scanner with parameters: tube voltage of 100–140 kV, tube current of 22–582 mA, section thickness of 0.5–1.0 mm, and pitch factor of 0.35–0.90.

In accordance with standard casework protocols, during original casework, PM data was compiled from conventional PM dental examination and reconciled with AM data compiled from dental records. Reconciliation resulted in a subjective assessment of the identification outcome using standard INTERPOL terms (excluded, insufficient data, possible ID, probable ID, or established ID) [4]. Following standard casework protocols, independent AM and PM data collation, and reconciliation by two FO operators ensured that peer review of the process and conclusion were undertaken.

In this study, PM data was generated for each case using only PMCT images. Two operators were utilized to simulate peer-reviewed processes—one experienced forensic odontologist and one post-graduate forensic odontology student. The operators had 2 years and 1 year of experience in viewing PMCT images respectively. Two-dimensional images and 3D reconstructions were viewed using the medical image viewer software Horos v4.0.0 RC5 (Nimble Co LLC d/b/a Purview in Annapolis, MD USA). Each PMCT scan was reviewed in 2D using anterior and lateral views and a curved MPR image. Three-dimensional image assessment was carried out by selecting 3D volume rendering. All PMCT data was viewed on high-resolution computer monitors without natural or artificial lighting besides that generated by the computer monitor.

Operators independently completed PM charting using the FDI World Dental Federation tooth numbering system [17] and recorded PMCT features using INTERPOL DVI dental codes [4]. Following charting from PMCT images, each operator completed reconciliation by reviewing against all original AM data. As per standard casework protocols, operators independently reached an identification outcome, then discussed each case to reach a consensus on both the final PM charting and the identification outcome.

PMCT charting and reconciliation outcomes were compared with those originally reached using conventional examinations, with differences between the two methods assessed.

Charting differences were classified as follows:

- Meaningful: may result in an exclusionary point or impact the reconciliation decision-making process. Examples include missed or additional features charted or additional or deficient surfaces for correctly charted features.
- Trivial: not considered likely to have any impact on the reconciliation decision-making process. Examples include instances of transposition, minor nomenclature differences, or correctly charted features with insufficient detail.

The quality of the remains on PMCT and evidentiary value of AM datasets were assessed.

### PM remains

- Categorized as:

- Intact – no injuries to jaws or teeth
- Fragmented – broken jaws and/or teeth, including avulsed teeth and fragments
- Incomplete – some jaw fragments and/or teeth not available for examination

### AM datasets

- Subjective scoring – opinion only:

- Poor
- Fair
- Good

- Objective scoring – As there is no currently accepted method for scoring of evidentiary value, we devised a formula using variables that have anecdotally been shown to be important. This formula reflects the importance of individualizing features and the presence of images by adding a multiplication factor to these variables.

- Weighted Score = *r* + *c* + *o* + 2*R* + 3*i.*

▪ *r* = Recency of creation to date of death: 0 = Not recent (5 or more years), 1 = Recent (less than 5 years)
▪ *c* = Clinical notes: 0 = none, 1 = limited, 2 = comprehensive
▪ *o* = Odontogram: 0 = none, 1 = partial, 2 = comprehensive
▪ *R* = Presence of imaging data: 0 = none, 1 = single area of dentition seen, 2 = multiple regions of dentition seen, 3 = complete dentition seen
▪ *i* = Individuality: 0 = less than 3 features, 1 = 3-10 features, 2 = greater than 10 features

- Final scores were categorized as follows:

▪  < 10 = Poor
▪ 10–14 = Fair
▪  > 14 = Good

- The final evidentiary value was calculated as the average of subjective and objective determination of Poor, Fair or Good.

The data were analyzed using descriptive statistics to determine factors that may influence variation in reconciliation outcome between the two methods of PM dental examination.

## Results

In this study, six of the 21 cases (28.6%) had a different reconciliation outcome when using PMCT alone. For these six cases when completed by conventional examination, four had an established reconciliation outcome, while two had a probable outcome. Based on PMCT data alone, the four established cases had probable outcomes, and the two probable cases had possible outcomes. In these six cases, PM examinations completed using only PMCT provided less certain reconciliation results compared to those completed using conventional PM dental examinations. The reconciliation outcomes for each case and each method of PM dental examination are detailed in Table 1.

A further analysis was performed to explore what contributed to these differences in reconciliation outcomes by evaluating the quality of AM data for each identified case, the condition of the remains for each PMCT, and the differences in PM charting between the two methods of PM dental examination.

The results of AM data assessment are detailed in Table 2. Overall, there was good agreement between subjective and objective assessments of the evidentiary value of AM datasets.

**Fig. 1:**
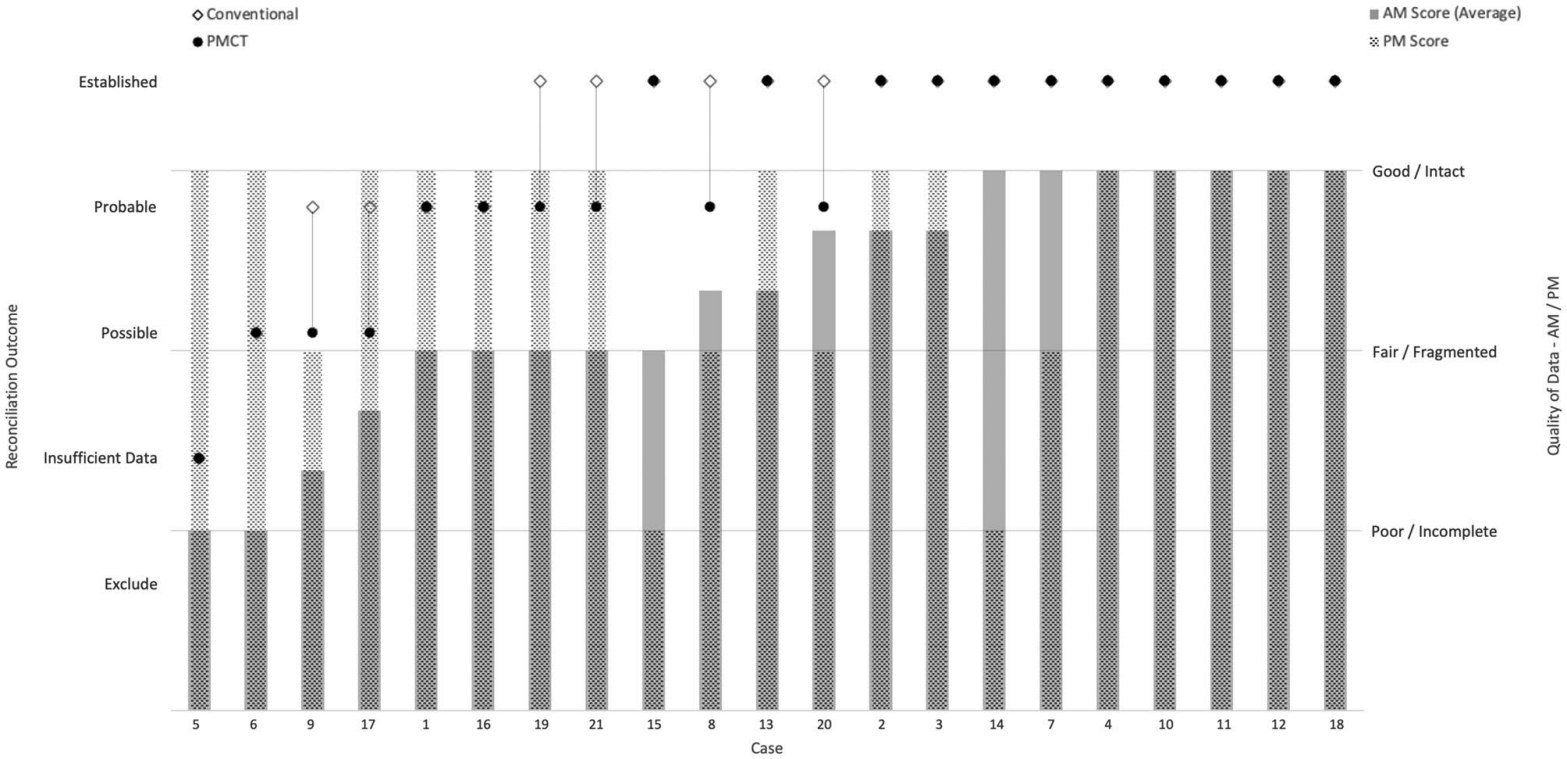
Scoring of the quality of AM and PM data compared with reconciliation outcomes—cases are organized into ascending quality of AM data. The quality of AM data plotted for each case is an average of the values obtained from operator 1, operator 2, and the objective scoring method

Based on the comparison of AM data quality with the reconciliation outcomes, Fig. 1 shows that confidence in establishing identity increased as the quality of the AM data improved. Differences in reconciliation outcomes occurred in half of the cases where AM data quality was assessed as above “Poor” and below “Good.” This suggests that when AM data quality is considered fair, a conventional PM dental examination is more likely to yield higher certainty of identification than a PMCT-only examination.

Figure 1 demonstrates that more intact PM datasets assisted in establishing identity when the evidentiary value of the AM datasets was between fair and good. Otherwise, there was little correlation between reconciliation outcomes achieved by PMCT examination and the quality of PM datasets.

When charting was compared for each method of PM dental examination, it was found that charting discrepancies existed for all cases included in this study. Complete results from the analysis of charting discrepancies are detailed in Table 3. A specific example of a meaningful charting difference is a tooth-colored restoration on the vestibular surface of the upper right second premolar identified from conventional examination versus the same tooth being marked as present and unrestored from PMCT examination.

When considering charting differences as they relate to the occurrence of discrepancies in reconciliation outcome between the two methods of PM dental examination, the highlighted cases in Table 3 show that discrepancies in reconciliation outcome occurred for cases with the number of meaningful charting differences ranging from 0 to 15. Overall, this indicates that charting differences did not correlate with the occurrence of discrepant reconciliation outcomes between the two methods of PM dental examination.

## Discussion

The aim of this study was to determine if there was an impact on reconciliation outcome when the collection of PM dental data was completed using only PMCT compared to conventional PM dental examination. The secondary aim was to assess factors that may contribute to differences in reconciliation outcomes between the two methods of examination. This study found that reconciliation outcomes differed for six out of the 21 cases examined, with conventional examinations achieving equal or greater certainty in establishing identity than PMCT examination. Assessment of potential factors driving these differences identified that the evidentiary value of AM data played a more significant role compared to the quality of PM data or the differences in PM charting between the two methods of PM dental examination. This highlights that the process of reconciliation is more complex than simply matching the three letter codes recorded on the odontograms and relies more on comparison of the details largely available in the antemortem and postmortem images.

Identification casework in FO relies on the ability to compare and reconcile AM and PM datasets; it is not simply a pattern-matching exercise. It has been demonstrated that clarity and strength of available information increase confidence in decision-making [18, 19]. Hence, it is likely that the evidentiary value and the quality of both AM and PM datasets would directly impact the reconciliation process and the certainty with which FOs are able to establish identity. In this study, reconciliation outcomes between the two methods of PM dental examination were concordant for two-thirds of the cases. Differences in outcome only occurred when the evidentiary value of AM data was deemed better than “Poor” but less than “Good.” The significance of this is that when fewer individualizing features are identified in AM data, a FO is more likely to be able to clearly identify and compare the corresponding features using a conventional means of PM dental examination than from PMCT examination. The likelihood of choosing “Established” as the final outcome was also influenced by the quality of the AM data. When the evidentiary value of AM datasets is “Good,” then it is possible to establish identity using either method of PM dental examination. When it is better than “Poor” but less than “Good,” then it is more likely to establish with conventional PM examination. However, neither method will reach an established outcome when the evidentiary value of AM datasets is considered poor.

Case 15 appears to have been the exception to this trend where identity was established using both PM examination methods despite the evidentiary value of the AM dataset being considered fair. Potentially, this is a reflection on the accuracy of our novel evidentiary value scoring system. Currently, available literature discusses how AM records are utilized in FO identification casework [20, 21] and the importance of improving the forensic relevance of dental records when created [22, 23]. However, there is a gap in the literature regarding how to objectively assign evidentiary value to AM records. For case 15, the individuality demonstrated in the AM dataset was high despite the records lacking recency, extensive clinical notes, or radiographic images covering extensive regions of the dentition. The individualizing features that were identified in the AM dataset included a unique pattern of missing and present teeth, a retained root in the upper left quadrant, and the pattern of restorations. There were only two intraoral radiographs available in the AM dataset, and one allowed clear visualization of the upper left jaw demonstrating the retained root, a root filling, and multiple restorations. These interesting features were readily identified and reconciled between AM and PM datasets to result in identity being established for this case. This case suggests that the presence of individualizing features, particularly if demonstrated in images, should be weighted more heavily in any future attempts to develop an objective method of scoring the evidentiary value of AM datasets.

In FO identification casework, reaching the final decision on identity is highly subjective. As alluded to by Chiam et al. (2022) [24], the reconciliation process and decision on identification outcome are not merely an exercise in matching dental patterns between AM and PM charts but also involve consideration of the reliability of datasets and the context in which it is presented. From the perspective of confidence in identification outcome, our study found that operators were more certain in establishing identity after conducting a conventional PM dental examination. This is most likely a reflection of the well-established difficulties associated with the discrimination of restorative materials, identifying restoration surfaces, and obstruction of features by metallic artifacts on PMCT images [14–16]. Another factor that may affect certainty in charting is the FO operator’s level of experience in reading PMCT—for example, experienced operators may find it easier to identify the presence of restorations and the surfaces involved on PMCT images compared to inexperienced operators. Studies have found that confidence in establishing identity is associated with the level of difficulty of a case [25, 26]. Likewise, this study shows that the general perception of less reliable information derived from PMCT resulted in less certain identification outcomes. Interestingly, this study also found that the number of charting discrepancies did not seem to influence the identification outcomes. This supports the concept that the final opinion is more reliant on the comparison of available images rather than on the matching of the charted codes.

## Conclusion

Based on the cases examined in this study, it was found that both conventional and PMCT methods of PM dental examination can reach similar identification outcomes. However, operators remained more certain in establishing identity when conducting PM dental examination by conventional means. This was most apparent when AM data was less than ideal. When AM data was either good or poor, then no differences were noted in operator certainty despite the PM data collection method. Given these findings, further research is required to identify cases suitable for completion utilizing PMCT alone and those that are not. Additionally, the development and validation of a more objective method for assessing the evidentiary value of AM datasets would be valuable. By investigating the impact on identification outcomes when using PMCT images, this study contributes to further validating the use of this technology in forensic odontology identification casework.

## Key Points

1. PMCT is currently not recommended as a standalone tool for PM dental data collection due to reduced accuracy in PM charting when compared with conventional PM dental examination.
2. Reduced accuracy when charting from PMCT is widely accepted, but the impact on the final identification outcome is yet to be explored.
3. This study compared identification outcomes achieved from PMCT examination with those from conventional PM dental examination.
4. Both methods of PM dental data collection can reach similar identification outcomes, but conventional PM dental examination yielded more certain identification outcomes than PMCT examination.
5. The quality of AM datasets is a strong predictor for the confidence in establishing identity.

**Table 1:**
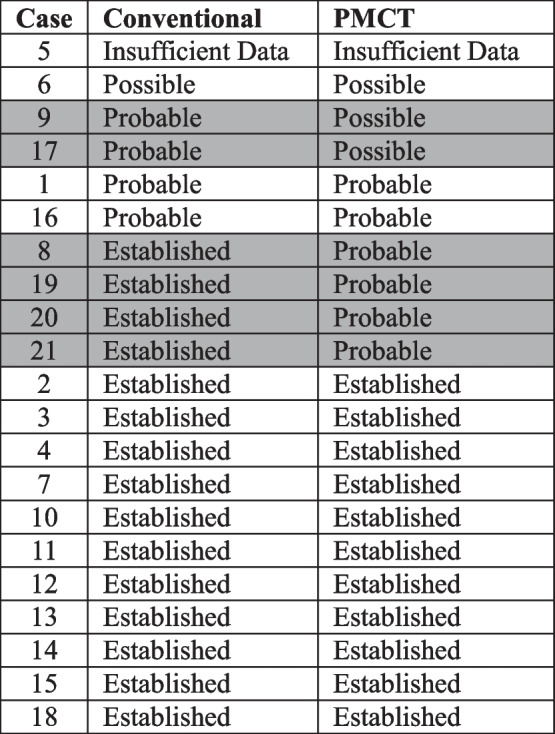
Reconciliation outcomes—cases are organized into ascending confidence in reconciliation outcome. The six highlighted cases are those with a discrepancy in reconciliation outcome between the two methods of PM dental examination

**Table 2:**
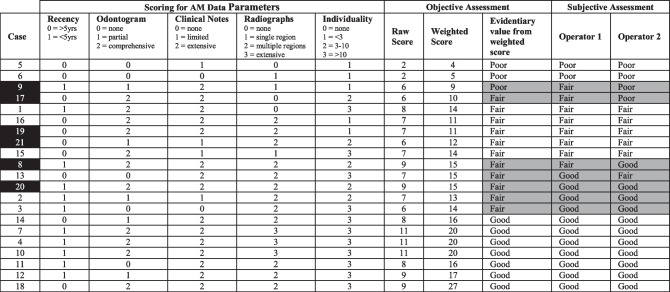
Evidentiary value of AM data—objective scoring and subjective assessment by operators 1 and 2. Cases are organized into ascending quality of AM data. The six cases highlighted in black are those with a discrepancy in reconciliation outcome between conventional and PMCT methods of examination. The seven cases with evidentiary values highlighted in grey are those with a discrepancy in the assessed quality of AM data between operator 1, operator 2, or the objective scoring method

**Table 3:**
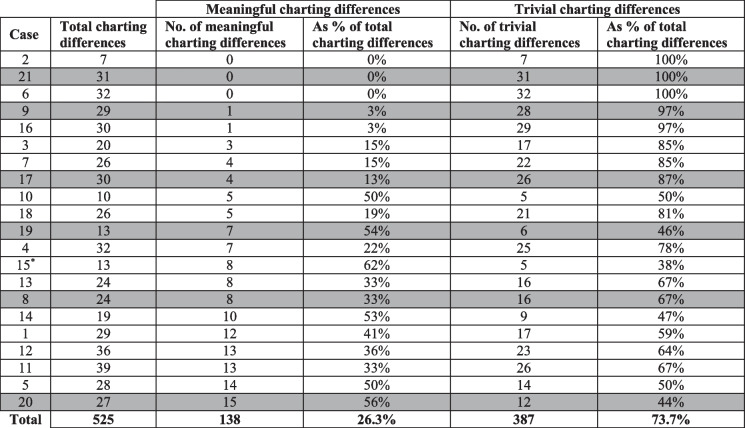
Differences in PM charting between the two methods of PM dental examination—cases are organized into ascending number of meaningful charting differences for each case. Cases highlighted in grey are those where there was a difference in reconciliation outcome between the two methods of PM dental examination. *Missing mandible on PMCT for case 15 so charting differences were only compared for maxillary teeth
